# From dispenser to nest: collection of fumigated material repels parasites across behavioural traits in Darwin’s finches

**DOI:** 10.1186/s40850-025-00250-2

**Published:** 2025-12-10

**Authors:** Lauren K. Common, Sonia Kleindorfer, Andrew C. Katsis, Katherine Albán Morales, Dominique M. E. Quirola, Birgit Fessl

**Affiliations:** 1 Konrad Lorenz Research Center for Behavior and Cognition, core facility of the University of Vienna, Grünau im Almtal, 4645 Austria; 2 Department of Behavioral and Cognitive Biology, University of Vienna, Vienna, 1030 Austria; 3 College of Science and Engineering, Flinders University, Bedford Park, SA 5042 Australia; 4 Charles Darwin Research Station, Charles Darwin Foundation, Puerto Ayora, Santa Cruz, Galápagos, Ecuador

**Keywords:** Avian vampire fly, *Philornis downsi*, Passeriformes: thraupidae, Invasive species control, Ectoparasite, Conservation

## Abstract

In the midst of a biodiversity crisis, it is important to conserve diversity at all levels, including species, genetic, and behavioural diversity. Animals with different behavioural traits can interact with their environment in distinct ways; hence, the distribution of behavioural traits within a population can influence the efficacy and outcome of conservation interventions. On the Galápagos Islands, Darwin’s finches are threatened by an invasive parasite, the avian vampire fly, *Philornis downsi*. Avian vampire fly larvae develop in the base of the nest, where they feed on the blood and tissue of developing nestlings, causing high mortality. In this study on Floreana Island, we deployed dispensers filled with insecticide-treated nesting material, a conservation method that has been found to decrease parasite load and increase fledging success. We then assessed whether individual differences in Darwin’s finch neophilia (response to a novel object) and aggressiveness (response to a simulated territory intrusion) affected their use of treated material. We found that ~57% of nests contained treated nesting material and that increasing volumes of treated material were associated with decreased parasite load. We found no effect of neophilia or aggressiveness on the probability of using treated material or amount of treated material used. Our results support nesting material dispensers as an effective conservation measure that conserves the full range of behavioural responses. To increase the efficacy of this method, future research should consider species- and individual-level preferences for material type and dispenser location.

**Supplementary Information:**

The online version contains supplementary material available at 10.1186/s40850-025-00250-2.

## Introduction

Anthropogenic activity has accelerated species extinction rates to levels comparable to the five previous mass extinctions [1, 2]. There is a pressing need to conserve and restore biodiversity globally, both for ecosystem functioning [3] and for human health [4, 5]. Outside of species diversity, it is important to conserve diversity on multiple levels. Genetic diversity is linked to evolutionary fitness and ecological processes such as competition, community structure, and primary productivity [6, 7]. Animals also have rich cultural and behavioural diversity that can affect population structure and sexual selection (e.g. [8]). Therefore, cultural and behavioural diversity is worthy of conservation, and may affect the viability of conservation interventions [9].

Animal behaviour interacts with wildlife management across multiple levels and the relatively young field of conservation behaviour has grown rapidly [10, 11]. Certain behaviours may increase the susceptibility of species to threats [12–14] or decrease effective population size [15]. The behaviour of species or populations that are affected by, or are the target of, conservation efforts may alter the efficacy of conservation methods. For example, if prey do not rapidly reinstate anti-predator strategies after native predators are reintroduced to an ecosystem, the reintroductions would negatively impact biodiversity. However, Berger [16] found that prey return to normal anti-predator behaviour within one generation of reintroduction. Behaviour at the species, population, and individual levels may each affect conservation outcomes [10].

Although the importance of individual behavioural differences for conservation has been recognised for nearly two decades [11], integrating this knowledge into conservation practices has been slow [17–19]. To date, most research into the effects of behavioural differences on conservation interventions has focused on captive breeding and rearing programs [20, 21] and translocations [22–24]. For example, more neophilic swift foxes, *Vulpes velox*, were more likely to die early following reintroduction to the wild [25]. Conversely, more exploratory desert tortoises, *Gopherus agassizii*, were more likely to survive post-translocation [26], which suggests that these patterns can be species-specific. Most studies assessing the role of behavioural differences in conservation programs have measured individual behaviour during short- or long-term captivity [18], while less is known about this interaction when conservation interventions are applied to wild animals in situ [27].

The current greatest threat to the conservation of the iconic Darwin’s finches (subfamily Geospizinae) on the Galápagos Islands is the invasive avian vampire fly, *Philornis downsi* (Diptera: Muscidae) [28, 29]. Adult avian vampire flies, although vegetarian, lay their eggs in the base of the nests of altricial birds [30]. Their larvae are parasitic, feeding on the blood and tissue of developing nestlings both internally and externally [31]. Parasitism by the avian vampire fly can have severe short- and long-term impacts, including blood loss, decreased growth, permanent deformation and high mortality [30], [32–34]. Host mortality is particularly high in the medium tree finch, *Camarhynchus pauper* [35], and mangrove finch, *C. heliobates* [36, 37], both of which are critically endangered. Knutie et al. [38] demonstrated that Darwin’s finches readily collected nesting material from dispensers and could successfully ‘self-fumigate’ their nests by integrating insecticide-treated nesting material, significantly reducing parasite load. Since then, this method has successfully been applied to the forty-spotted pardalote, *Pardalotus quadragintus,* in Australia [39] and other Darwin’s finch species on the Galápagos Islands [40, 41]. This method is relatively cost- and time-effective and requires less labour than alternative techniques, such as locating and treating individual nests, making it a prime candidate for more widespread use. However, in the four studies mentioned above, not all candidate nests were found to contain insecticide-treated nesting material [38–41]. Differences in behavioural traits may drive parents’ ability or willingness to utilise this resource and thereby reap the benefits of decreased parasite intensity.

To reduce the parasite load in Darwin’s finch nests on Floreana Island, we deployed 90 dispensers filled with different types of insecticide-treated nesting material during the breeding season. However, individuals may differ in their use of these dispensers, which may affect their parasite load and, therefore, their reproductive success. We ask: 1) Does insecticide-treated nesting material reduce the number of avian vampire flies in the nest? We predict that the total number of avian vampire flies will decrease with increasing amounts of insecticide-treated nesting material integrated into the nest. 2) Does the behaviour of nest owners affect the presence and amount of treated nesting material integrated into their nest? We predict that i) more neophilic individuals (more likely to approach and interact with novel objects) will be more likely to use treated nesting material in their nest than less neophilic individuals; and ii) more aggressive males (responding more strongly to a simulated territory intrusion) have a higher probability of using treated nesting material, as they are more likely to defend novel resources and deter less aggressive conspecifics from their territory.

## Methods

### Study sites

This study was conducted during the 2024 Darwin’s finch breeding season (January–March) at four sites on Floreana Island, Galápagos Archipelago, Ecuador. Two of our sites were in the humid highlands (300–400 m asl) and two in the arid lowlands (0–150 m asl). The highland sites, Cerro Pajas (01° 17’ S, 090° 27’ W) and Asilo de la Paz (01° 18’ S, 090° 27’ W), are primarily endemic *Scalesia* forest. The lowland sites were the town of Puerto Velasco Ibarra (01° 16’ S, 90° 29’ W), which has a population of approximately 160 people [42], and the surrounding uninhabited areas of scrubland (01° 17’ S, 90° 28’ W) [43].

We monitored four species of Darwin’s finch (Passeriformes: Thraupidae): the common cactus finch (*Geospiza scandens)*, small ground finch (*G. fuliginosa*), small tree finch (*Camarhynchus parvulus)* and medium tree finch (*C. pauper)*. Darwin’s finches are socially monogamous per nesting attempt. Males build one or more domed display nests and sing at the nest to attract a female. Once a female has chosen a male, either the female lines the existing nest or the pair build a new one together [44]. For the structure of their nests, Darwin’s finches use a variety of nesting materials, primarily moss, leaves, grass, thin sticks, and seeds (such as the endemic Darwin’s cotton, *Gossypium darwinii* [45]). They will then line their nest with feathers, fine grass, plumed seeds, and/or fur. Darwin’s finches are known to integrate artificial material into their nest [46], including insecticide-treated cotton when dispensers are installed nearby [38, 40, 41].

### Nesting material dispensers

**Fig. 1:**
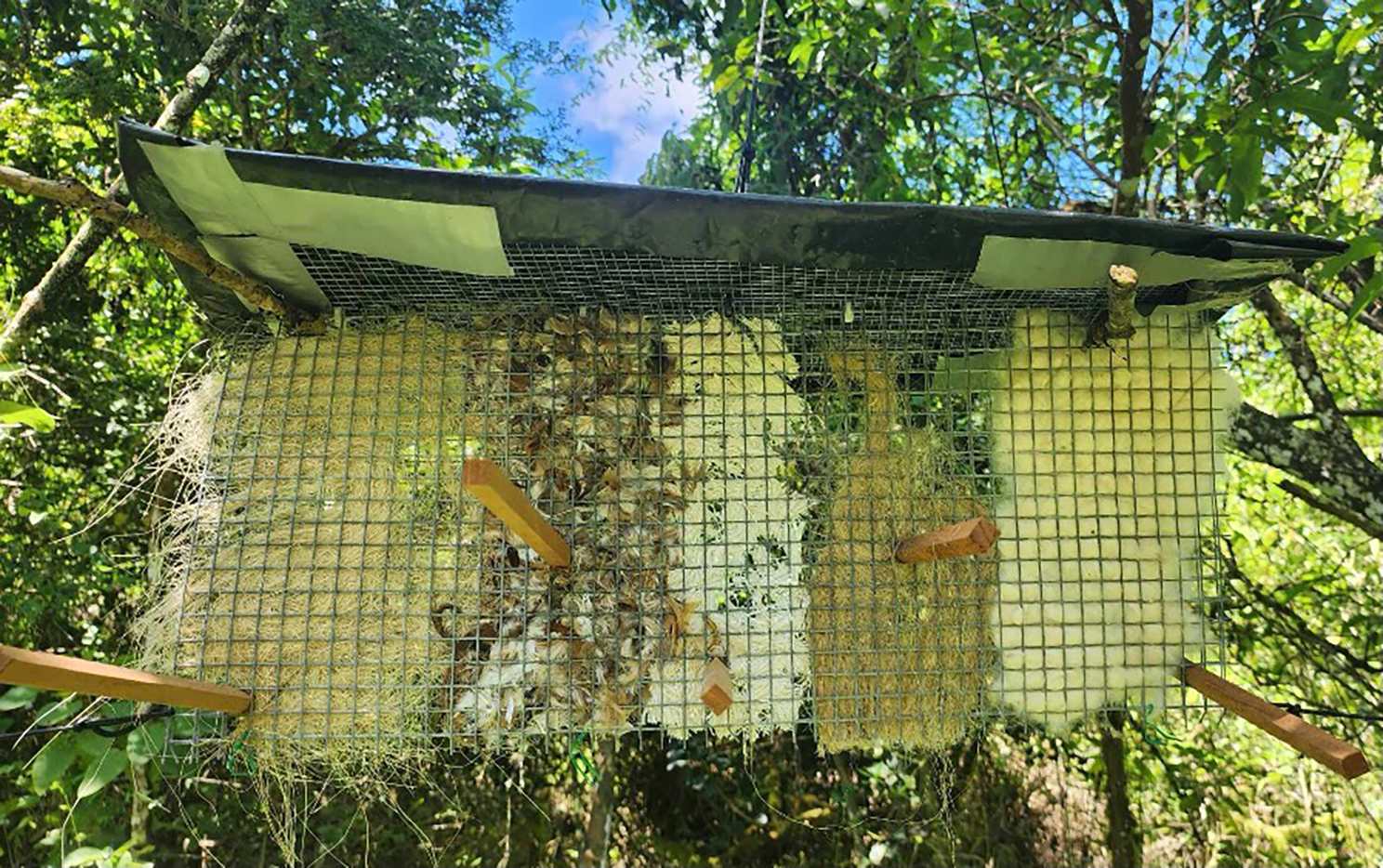
An insecticide-treated nesting material dispenser deployed at Cerro Pajas, Floreana Island, Galápagos. The materials from left to right are: sisal fibres, feathers, cotton fibres, hemp fibres, and kapok

We deployed 90 nesting material dispensers across two sites on Floreana: Cerro Pajas (50 dispensers) and the lowlands (40 dispensers). Dispensers were placed at 1.5–3 m high along transects, separated by 50 m. Each dispenser contained five different materials: sisal fibre (12 g), cotton fibre (12 g), chicken feathers (8 g), kapok (4 g) and hemp fibre (6 g), for a total of 42 g of material per dispenser (Fig. 1). Different masses were selected for each material to ensure approximately equal volumes per dispenser. All dispenser material was thoroughly sprayed with 1 mL of 0.5% PermaCap CS controlled release insecticide solution (BASF United States) per 1 g of material (21 mL on the front, 21 mL on the back; 42 mL total) [40, 41, 47]. Dispensers were refilled and resprayed with 0.5% PermaCap every two weeks to ensure the insecticide’s efficacy. The dispensers were removed after seven weeks, at the end of the field season.

### Nest monitoring and dismantling

Following long-term protocols, we monitored Darwin’s finch nesting activity at all four sites, including those without nesting material dispensers [48]. Territories and nests were monitored every three days during defending, pairing, and incubation, and every two days during feeding. Observations were undertaken between 0600 and 1200 GALT to minimise time-of-day effects. To confirm breeding activity, nests were checked either with a borescope or by observing parental behaviour using binoculars. Upon termination of the nesting attempt (either because the chicks fledged or the nest failed), nests were collected and placed in plastic ziplock bags and dismantled within 24 hours. Male display nests that were not selected by females were also collected after confirming abandonment by the male (i.e. the male was not seen near the nest during 30-min observations across at least 2 days). We only collected the nests of three species (small ground finch, small tree finch, and medium tree finch), as all monitored cactus finch nests were still active at the end of the field season. We counted the number of avian vampire fly larvae, pupae, and empty puparia to determine total parasite load. If the nests were wet, they were placed under a heat lamp for 8–24 hours until dry. Nesting material was then divided into natural material and treated material from the dispensers (sisal fibre, cotton fibre, chicken feathers, kapok and hemp fibre). Each material type was weighed to the nearest 0.01 g using a digital scale (TANITA 1479J2 Digital Precision Pocket Scale, TANITA Europe B. V.) and volume was calculated using the known densities of each fibre (Table S1 [41]). The volumes of all five materials were summed to calculate total volume of treated material for each nest (mm^3^). In total, we dismantled 96 nests across the four study sites.

### Neophilia – novel object trials

We conducted novel object trials as a measure of neophilia in male and female nesting Darwin’s finches across all sites. We conducted trials on 103 individuals at 81 nests from four species (cactus finch = 5 nests, small ground finch = 24 nests, small tree finch = 29 nests, medium tree finch = 23 nests). Trials were conducted before eggs were laid (i.e., during defending and paired stages), as these are the main nest-building stages. First, a novel object was placed 5 m from the nest (diagonally), at least 1 m high, visible from the nest entrance and hanging freely in the vegetation. Once placed, the experimenter stood 10 m away and a bird whistle (Audubon Bird Call) was used continuously for 1 min or until a focal individual appeared within 5 m of the novel object [49]. This device creates a squeaking sound that should be novel to the study individuals [49]. The call does not sound like any local bird species and does not appear to elicit an aggressive response, as individuals do not sing, display, or fly across aggressively when it is played. Focal individuals, i.e. the nest owners, were determined based on their behaviour (defending the nest, displaying near the nest, or building the nest) and the amount of black in their plumage (males of both the same species and grade of black plumage rarely have adjoining territories) [50]. The trial began once a focal bird entered within 5 m of the novel object and lasted 5 mins. If a focal individual did not appear after 10 mins, the bird whistle was repeated for 1 min. Four novel objects were used: a yellow-and-orange tape measure, multicoloured ukulele, blue dry bag, and red-and-grey expandable strainer (Fig. S1). Objects were randomised per nest. Each individual was tested once. Although the individual consistency of object neophilia has not been demonstrated in Darwin’s finches, previous work in this study population found a near-significant positive among-individual correlation between object neophilia and exploration of a novel environment—which is itself a significantly repeatable trait [49]. All novel object trials were conducted between 0600 and 1200 GALT and scored by a single experimenter (LKC). The variables extracted were latency to within 3 m of the novel object (in secs) and minimum distance from the novel object (in m).

### Aggressiveness – territory defence trials

To measure individual aggressiveness, we conducted territory defence trials using song playback across all sites. Aggressiveness is a consistent and repeatable behavioural trait in male Darwin’s finches on Floreana Island [51, 52]. Trials were conducted before eggs were laid (defending and paired stages). We conducted trials at 58 nests (cactus finch = 5 nests, small ground finch = 19 nests, small tree finch = 13 nests, medium tree finch = 21 nests). We analysed each individual’s first trial. We only conducted playback trials for male nest owners, as female Darwin’s finches rarely respond to playback [53]. We constructed our playback stimuli using Darwin’s finch songs recorded on Floreana Island in 2023. These songs were recorded as uncompressed 16-bit broadcast wave files (.wav) using a Sennheiser ME67 directional microphone (Sennheiser electronic GmbH & Co., USA) connected to either a Marantz PMD 660 Solid State recorder (Sound United, LLC, USA) or a Zoom H6 recorder (Zoom North America, USA). We created 25 unique playback tracks (8 small ground finch, 8 small tree finch, 7 medium tree finch, 2 cactus finch), each lasting 3 min (1 min of song playback, 1 min of silence, 1 min of song playback). Each 1-min song playback period contained six repetitions of the same male song type, simulating a territory intrusion by a single unfamiliar conspecific. We used a high-pass filter to remove sounds < 1 kHz and saved the playback tracks as uncompressed 16-bit wave files, which were then transferred to an Apple iPod (Apple Inc., USA). Songs were broadcast in the field using a Sony XB12 Extra Bass Portable Bluetooth Speaker (Sony Australia Limited), with a frequency response of 20 Hz–20 kHz. Playback tracks were selected randomly, while ensuring that the track matched the species of the focal individual. The speaker was placed at 1–1.5 m high and we only began playback when the male was seen within 20 m of the speaker. All territory defence trials were conducted by one of two experimenters (ACK or SK) between 0600 and 1200 GALT and were scored by a single experimenter (ACK). The extracted variables were time spent within 5 m of the speaker (in secs), time spent within 1 m of the speaker (in secs), minimum distance to the speaker (in m), number of flights, and number of crosses (flights that cross the speaker) [52].

### Statistical analysis

All statistical analyses were conducted in R version 4.5.0 [54]. Linear models (LMs) and generalised linear models (GLMs) were performed using the ‘lm’ and ‘glm’ functions, respectively, in stats package version 4.5.0. Linear mixed models (LMMs) and generalised linear mixed models (GLMMs) were performed using the ‘lmer’ and ‘glmer’ functions in the package lme4 version 1.1–33 [55].

#### Principal component analysis

We used two principal component analyses (‘prcomp’ function in stats package) to reduce our behavioural response variables, scaled within the ‘prcomp’ function, to a single measure of neophilia (PC_Neophilia) and a single measure of aggressiveness (PC_Aggressive). These variables were used to assess which factors influence neophilia and aggressiveness at the trial level. The neophilia analysis included latency to 3 m and minimum distance to the novel object (matrix rotation 2 × 2); the first principal component had eigenvalue 1.82 and explained 91% of variation. We took the inverse values for PC_Neophilia so that high scores indicated greater exploration towards the novel object, i.e., shorter latency to approach within 3 m and shorter minimum distance. The aggressiveness analysis included time within 5 m, time within 1 m, minimum distance to speaker, number of flights, and number of crosses (matrix rotation 5 × 5); the first principal component had eigenvalue 3.16 and explained 63% of variation. We took the inverse values for PC_Aggressive so that high scores indicated a more aggressive response towards the simulated territory intrusion, i.e., more time spent within 5 m and 1 m of the speaker, shorter minimum distance to the speaker, and more flights and crosses. Factor loadings for all PCs are available in Table S2.

#### Factors associated with neophilia and aggressiveness

To determine which factors are associated with neophilia and aggressiveness in Darwin’s finches, we ran two separate models. The first model (LMM) had PC_Neophilia as the dependent variable, with sex, breeding stage (defending, paired), site (lowlands, Cerro Pajas and Asilo de la Paz), species (four levels: cactus finch, small ground finch, small tree finch, and medium tree finch), and novel object type as fixed effects. We included nest ID as a random effect because both the male and female were tested at 22 nests. We initially included an interaction between species and novel object type to investigate species preferences; however, this term was non-significant and subsequently removed from the model. The second model (LM) had PC_Aggressive as the dependent variable, with breeding stage, site, and species included as fixed effects. Because aggressiveness was only measured for one member of the breeding pair (i.e. only male nest owners), this model did not include nest ID as a random effect.

#### Neophilia, aggressiveness, and treated material use

To determine if use of treated material differed between species (three levels: small ground finch, small tree finch, and medium tree finch), site, and distance to the nearest dispenser, we ran two models on the subset of nests that were within 100 m of a dispenser and were built after the dispensers were deployed (*N* = 44 nests, 40 highlands and 4 lowlands). The first model was a binomial GLM testing whether the nest contained any amount of treated material (0 = no, 1 = yes). The second model was a linear model testing which factors influenced the volume of dispenser material (in mm^3^, log-transformed to fulfill the assumption of normality). Both models included species, site, and distance to the nearest dispenser as fixed effects. We used the package ‘emmeans’ version 1.7.1–1 [56] to calculate post hoc pairwise comparisons with Tukey *p*-value adjustment for significant species effects.

To determine if parental neophilia predicted nesting material use, we ran two separate models: (1) a binomial GLMM with the presence of treated material (0 = no, 1 = yes) as the dependent variable, and (2) a linear model with the volume of dispenser material (log-transformed) as the dependent variable. Both models included PC_Neophilia and distance to the nearest dispenser as fixed effects. We originally included sex and species as fixed effects, but they caused model convergence and fit issues and were, therefore, removed from the final model. Only nests that were 100 m or less from the nearest dispenser were analysed. At 6 of these nests, we had neophilia data for both members of the breeding pair; consequently, we included nest ID as a random effect in our first model to control for the non-independence of these values. We initially included nest ID as a random effect in the second model, but the model did not converge and so the random effect was omitted.

To determine if paternal aggressiveness predicted nesting material use, we ran two separate models: (1) a binomial GLM with the presence of treated material (0 = no, 1 = yes) as the dependent variable, and (2) a linear model with the volume of dispenser material (log-transformed) as the dependent variable. Both models included PC_Aggressive, distance to the nearest dispenser, and species (three levels) as fixed effects. Nest ID was not included as a random effect, as we tested only males within each breeding pair.

To assess whether the amount of treated material influenced the number of avian vampire flies in the nest, we conducted a GLM using the subset of nests that reached the chick stage (*N* = 26 nests), as no avian vampire flies were found in nests that failed during incubation. We used a negative binomial distribution, as the model was significantly overdispersed when assuming a Poisson distribution (testDispersion function in DHARMa, dispersion parameter = 23.5, *p* < 0.001). Dispersion was not significant when assuming a negative binomial distribution (dispersion parameter = 0.45, *p* = 0.488). We included nests from all study sites, including those that did not have dispensers, to accurately estimate the number of avian vampire flies at nests with no dispenser material. The model included the total number of avian vampire flies as its dependent variable, with species (three levels), site, and total volume of dispenser material as fixed effects.

Continuous variables in all models were scaled to facilitate the interpretation of effect sizes (standardised to mean = 0 and standard deviation = 1). Model assumptions and dispersion were assessed using the package DHARMa version 0.4.4 [57]. We extracted χ^2^ and *p*-values from the ANOVA Table of Deviance using Type III χ2 tests (‘Anova’ function in the package car [58]). We report model effect sizes as estimate ± standard error (SE) using the ‘summary’ function in lme4. Predicted values of models were extracted using the ‘ggpredict’ function in the package ggeffects version 1.2.3 [59], and all graphs were created using ggplot2 version 3.5.1 [60] and ggpubr version 0.4.0 [61].

## Results

### Factors associated with neophilia and aggressiveness

Neophilia was not predicted by site, species, nesting stage, or novel object type (*N* = 103; Table S3). Aggressiveness was not predicted by nesting stage or site (*N* = 58; Table S4); however, species differed significantly in their aggressiveness, with scores highest for the small ground finch (χ^2^ = 31.61, *p* = 0.012) (Table S4, Figure S2).

### Neophilia, aggressiveness and treated material use

Of the 44 nests collected from sites with dispensers in the lowlands (*N* = 4) and highlands (*N* = 40), 25 contained some amount of treated nesting material (~57%). The volume of treated nesting material ranged from 8 mm^3^ to 7276 mm^3^ (mean = 821.0 ± 336.8 mm^3^). All three species integrated treated nesting material into their nests (number of nests with treated material: small ground finch = 12 out of 18 nests, small tree finch = 7 out of 14, medium tree finch = 6 out of 13 nests). Species did not differ in their likelihood of using treated nest material (χ^2^ = 2.81, *p* = 0.245; Table 1a), although there was a non-significant trend for nests closer to a dispenser to be more likely to contain treated material (χ^2^ = 3.36, *p* = 0.067, Table 1a). The volume of treated material in a nest was inversely predicted by the distance to the nearest dispenser (χ^2^ = 6.27, *p* = 0.037) (Table 1b). There were no effects of site on either the probability of treated material being integrated in the nest or the volume of material in the nest (Table 1), although lowland sample size was low (*n* = 4). Species differed significantly in their volume of treated material integrated into the nest (F = 4.25, *p* = 0.022, Table 1b). Post hoc comparisons showed that small ground finches used more treated material than small tree finches (post-hoc estimate = 1.16 ± 0.44, *p* = 0.030), medium tree finches tended to use less than small ground finches (post-hoc estimate = −1.08 ± 0.46, *p* = 0.064), and small and medium tree finches did not differ from each other (post-hoc estimate = 0.09 ± 0.46, *p* = 0.981).

**Table 1:** The effects of species, site, and distance to the nearest dispenser on the a) probability of treated nesting material being integrated (binomial GLM) and b) volume (mm^3^, log-transformed, linear model) of treated nesting material in Darwin’s finch nests on Floreana Island (*N* = 44 nests). Species abbreviations: SGF = small ground finch, STF = small tree finch, MTF = medium tree finch. Bold values indicate significance *P* < 0.05

	***Estimate***	***SE***	***z-value***	***χ***^***2***^	***df***	***P***
*a) Probability of treated nesting material found in nests*						
Intercept	−0.18	0.60	−0.30			
Species [SGF]*	1.16	0.87	1.34	2.81	2	0.245
Species [STF]*	−0.09	0.81	−0.11			
Site [Lowlands]**	1.02	1.31	0.78	0.66	1	0.416
Distance to nearest dispenser	−0.72	0.48	−1.50	3.36	1	0.067
	**Estimate**	**SE**	**t-value**	**SumSq**	**F-value**	**P**
*b) Amount of treated nesting material found in nests*						
Intercept	0.83	0.34	2.45			
Species [SGF]*	1.08	0.46	2.33	11.47	4.25	**0.022**
Species [STF]*	−0.09	0.46	−0.19			
Site [Lowlands]**	0.48	0.64	0.74	0.74	0.55	0.463
Distance to nearest dispenser	−0.41	0.19	−2.16	6.27	4.65	**0.037**

^*^Species MTF set as reference category

^**^Site Cerro Pajas set as reference category

**Fig. 2:**
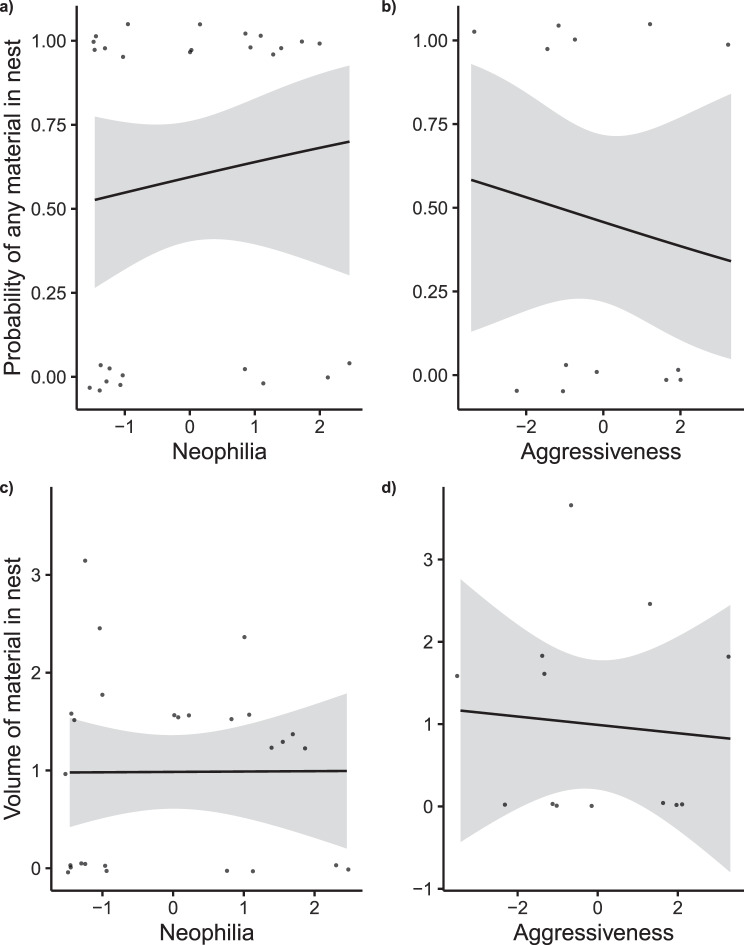
Raw and predicted values (regression line, black) showing the relationship between nest owners’ behavioural response (neophilia or aggressiveness) and their use of insecticide-treated nesting material. **a**,**b**) The probability that any amount of treated material was found in the nest (binomial, 0 = no treated material in nest, 1 = treated material in nest). **c**,**d**) The volume of treated material found in the nest (mm^3^, log-transformed). Neophilia (PC_neophilia) is an individual’s response to a novel object, with higher scores indicating greater neophilia. Aggressiveness (PC_aggressive) is an individual’s response to a simulated territory intrusion, with higher scores indicating greater aggressiveness. Raw data are presented as circles. Full model output presented in Table 2. Grey ribbons represent 95% confidence intervals around the regression line

The neophilia (*N* = 27 individuals) of nest owners did not predict the presence or absence of treated material in their nest, nor the volume of treated material (Table 2, Fig. 2). Similarly, the aggressiveness (*N* = 13 individuals) of nest owners did not predict the presence or absence of treated material in their nest, nor the volume of treated material (Table 2, Fig. 2).

**Table 2:** The relationship between a nest owners’ behavioural response (neophilia or aggressiveness) and its use of insecticide-treated nesting material in three species of Darwin’s finch. a) Binomial GLMM testing whether neophilia (response to a novel object, PC_Neophilia, *N* = 27 individuals) predicts the presence of treated material. Random factor nest ID explains 2015 ± 44.88 of the variance. b) Linear model testing whether neophilia predicts the volume of dispenser material found in the nest (mm^3^, log-transformed, *N* = 27 individuals). c) Binomial GLM testing whether aggressiveness (response to a simulated territory intrusion, PC_Aggressive, *N* = 13 individuals) predicts the presence of treated material. d) Linear model testing whether aggressiveness predicts the volume of dispenser material found in the nest (mm^3^, log-transformed, *N* = 13 individuals). Species abbreviations: SGF = small ground finch, STF = small tree finch, MTF = medium tree finch. Bold values indicate significance *P* < 0.05

	***Estimate***	***SE***	***z-value***	***χ***^***2***^	***df***	***P***
*a) Neophilia and probability of treated material in nest*						
Intercept	11.34	4.46	2.54			
PC_Neophilia	−0.61	2.16	−0.28	0.08	1	0.778
Distance to nearest dispenser	−6.49	2.72	−2.38	5.68	1	**0.017**
	**Estimate**	**SE**	**t-value**	**SumSq**	**F**	**P**
*b) Neophilia and amount of treated material in nest*						
Intercept	1.01	0.18	5.72			
PC_Neophilia	−0.05	0.18	−0.30	0.08	0.09	0.766
Distance to nearest dispenser	−0.29	0.17	−1.70	2.42	2.89	0.102
	**Estimate**	**SE**	**z-value**	**χ**^**2**^	**df**	**P**
*c) Aggressiveness and probability of treated material in nest*						
Intercept	−1.48	1.46	−1.01			
PC_Aggressive	−0.13	0.92	−0.14	0.02	1	0.891
Distance to nearest dispenser	−1.99	2.00	−1.00	2.15	1	0.142
Species [SGF]*	1.83	2.13	0.86	0.99	2	0.610
Species [STF]*	1.17	1.62	0.72			
	**Estimate**	**SE**	**t-value**	**SumSq**	**F**	**P**
*d) Aggressiveness and amount of treated material in nest*						
Intercept	0.96	0.76	1.27			
PC_Aggressive	0.06	0.60	0.10	0.02	0.01	0.921
Distance to nearest dispenser	−0.40	0.46	−0.86	1.52	0.73	0.417
Species [SGF]*	0.14	1.34	0.10	0.05	0.01	0.988
Species [STF]*	−0.08	1.06	−0.71			

^*^Species MTF set as reference category

**Fig. 3:**
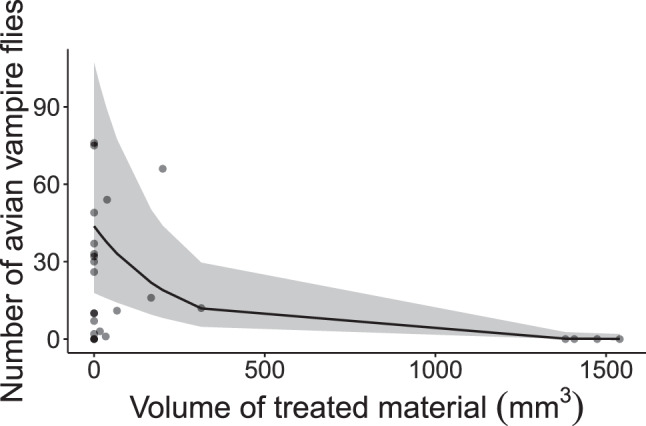
Raw and predicted values (marginal effect regression line, black) showing the relationship between the volume of insecticide-treated nesting material and the total number of avian vampire flies found in Darwin’s finch nests (*N* = 26). Raw data are presented as circles. Full model output presented in Table 3. Grey ribbon represents 95% confidence intervals around the marginal effect regression line

Across all four sites, when considering only nests that hatched chicks (i.e. those with the possibility of being infested with avian vampire flies, *N* = 26), the number of avian vampire flies significantly decreased with increasing volume of treated material (estimate = −0.004 ± 0.001, *p* < 0.001) (Table 3, Fig. 3).

**Table 3:** The effects of species, site, and volume of treated nesting material on the number of avian vampire flies in Darwin’s finch nests on Floreana Island (*N* = 26). Negative binomial GLM. Species abbreviations: SGF = small ground finch, STF = small tree finch, MTF = medium tree finch. Dispersion parameter for negative binomial (0.647) taken to be 1. Bold values indicate significance *p* < 0.05

	Estimate	SE	z-value	**χ**^**2**^	df	P
Intercept	2.56	0.90	2.85			
Species [SGF]*	1.08	1.03	1.05	1.73	2	0.421
Species [STF]*	0.35	1.11	0.32			
Site [Cerro Pajas]**	0.13	0.64	0.20	0.68	2	0.712
Site [Lowlands]**	−0.62	0.88	−0.70			
Treated material	−0.004	0.001	−3.56	15.79	1	**< 0.001**

^*^Species MTF set as reference category

^**^Site Asilo de la Paz set as reference category

## Discussion

During conservation programs, ensuring the maintenance of diversity within species—be it genetic, cultural, or behavioural diversity—is of utmost importance. In this study, we assessed whether an invasive parasite intervention differed in efficacy depending on the behaviour of individual birds. We found that use of insecticide-treated nesting material was relatively common across three Darwin’s finch species, with 57% of nests integrating treated material. Even small amounts of treated material were associated with a reduction in the number of invasive avian vampire flies. Encouragingly, the nest owners’ neophilia and aggressiveness did not predict the presence or amount of treated material in their nest. These results suggest that nesting material dispensers are used throughout the population regardless of behaviour, which may conserve the range of behavioural traits in Darwin’s finches. However, we found differences in the amount of material collected between species, with small ground finches using significantly more material than small tree finches and tending to use more than the critically endangered medium tree finch. Hence, increasing the rate of dispenser usage by medium tree finches may be critical for the species’ persistence.

Contrary to our predictions, the nest owners’ behavioural responses did not predict their use of treated nest material. This suggests, firstly, that neophilic individuals were not more likely to approach and utilise the nesting material dispensers. The dispensers were present in the environment for seven weeks, which may have been long enough for them to lose their novelty. Habituation to novel stimuli can be rapid (e.g. [62]), especially when exposure is more frequent [63, 64], and island species are particularly open to novelty [65, 66]. However, there may have been differences in how individuals interacted with the dispensers during the early period of their deployment that we were not able to capture within our study. Secondly, differences in male aggressiveness did not predict their use of treated nesting material. The dispensers were assigned at 50-m intervals, irrespective of territory owner or territory size, which varies between individuals and species [51]: therefore, not all birds had a dispenser in their territory. Presumably, those that happened to have a dispenser in their territory would have had access to it regardless of their aggressiveness. Conversely, if the dispenser was in their neighbour’s territory, access to nesting material may have depended on the neighbour’s aggressiveness rather than their own. In addition, we were also only able to measure male aggressiveness, since females, although known to defend their territory, rarely respond to song playback (Kleindorfer and Katsis, pers. obs.) [53]. Female Darwin’s finches perform the bulk of the nest lining [44]. This is the most important layer of the nest for treated nesting material, as the material types offered in the dispensers were similar to natural nest lining materials and avian vampire fly larvae are primarily in contact with the nest lining. Hence, the behaviour of the female may be more important for predicting treated material use than that of the male.

Although neophilia and aggressiveness did not predict material use, individuals still differed in their use of the material dispensers. We found that 57% of nests contained treated material, compared to 85% in Knutie et al. [38] and 84% in Alves et al. [39]. Material dispensers were closer together in Knutie et al. [38] (40 m apart compared to 50 m apart in our study) and directly beside the nest boxes in Alves et al. [39] (approximately 20 m apart). However, our results are similar to Mauchamp-Fessl [41] and Kofler et al. [40], both of which were conducted on Santa Cruz Island, where approximately 60% of small tree finch and green warbler finch, *Certhidea olivacea,* nests contained dispenser material, with the dispensers also placed 50 m apart. In our study, nests that were closer to a dispenser contained more treated nest material. Among the Darwin’s finches, treated material was found in nests up to 27 m away from the nearest dispenser, although another landbird species on the island, the Galápagos yellow warbler, *Setophaga petechia aureola*, collected treated material from more than 100 m away (Common et al. in prep). Reducing the interval between dispensers to 40 m (i.e., a radius of 20 m per dispenser) may increase usage but would significantly increase the cost and labour needed to deploy and maintain them. Therefore, more targeted placement of dispensers in established territories, such as those belonging to medium tree finches and mangrove finches, both island endemic species restricted to small patches of remnant forest, would be a reasonable approach [35–37, 67].

Although the results of this conservation strategy are promising, more research is needed to understand species- and individual-level differences in treated material use. All three studies to date, including this study, were restricted to one or two breeding seasons [38, 39, 41]. Therefore, long-term patterns of usage are not yet known. Similarly, there is currently no published information on the dispenser usage of other landbirds on the Galápagos that act as reservoir species for the avian vampire fly (e.g. the Galápagos mockingbird, *Mimus parvulus* [68]). Although we did not specifically test for differences in usage among the five material types, individuals and species may differ in their preference for certain materials based on their colour or structural properties [69 –71]. Indeed, Mauchamp-Fessl [41] and Kofler et al. [40] found preferences for certain material between Darwin’s finch species. Future research should investigate if other Darwin’s finch species, particularly the medium tree finch, differ in their preference for certain dispenser materials.

## Conclusion

We found that three species of Darwin’s finch integrated insecticide-treated nesting material into their nests, which was associated with reduced parasite load. We provide further evidence that dispensers filled with insecticide-treated material are an effective conservation measure against invasive avian vampire flies [38, 40, 41], but should be used in conjunction with other methods to significantly reduce the parasite population. Previous studies using paired experimental designs support the efficacy of this method for parasite reduction [38, 40]. We found no evidence that individual behaviour (neophilia or aggressiveness) was associated with the probability of using treated material or the amount of treated material found in the nest. Given that both aggressiveness [51, 52] and exploration [49] are consistent and repeatable across time in Darwin’s finches on Floreana Island, and that individual differences in behaviour can be heritable [72, 73], our findings suggest that this conservation intervention is not selecting for certain behavioural traits, and so conserves behavioural diversity in Darwin’s finch populations. However, we encourage further research into the long-term repeatability and heritability of behaviour in both Darwin’s finches and other species of conservation concern. Dispenser usage may be affected by other sources of individual variation, such as material preference [41, 69–71], or by the specific protocols used, such as distance between dispensers. Future research should investigate species and individual preferences for material type and location to increase the efficacy of this promising conservation strategy.

## Electronic supplementary material

Below is the link to the electronic supplementary material.

Supplementary Material 1

Supplementary Material 2

## Data Availability

Data are available on the University of Vienna data repository PHAIDRA: http://doi.org/10.25365/phaidra.625
