# Supplementary material for: From dispenser to nest: collection of fumigated material repels parasites across behavioural traits in Darwin’s finches
Source: BMC Zool. 2025 Dec 10;10:27. doi: 10.1186/s40850-025-00250-2 (PMC12703940; doi:10.1186/s40850-025-00250-2)
Supplement: Supplementary file 1 — Supplementary Material 1 [file 40850_2025_250_MOESM1_ESM.docx]

**Supplementary Information for**

**From Dispenser to Nest: Collection of Fumigated Material Repels Parasites Across Behavioural Traits in Darwin's Finches**

Lauren K. Common^1,2^, Sonia Kleindorfer^1,2,3^, Andrew C. Katsis^1,2^, Katherine Albán Morales^1,2^, Dominique M. E. Quirola^4^, Birgit Fessl^4^

^1^ Konrad Lorenz Research Center for Behavior and Cognition, core facility of the University of Vienna, 4645 Grünau im Almtal, Austria

^2^ Department of Behavioral and Cognitive Biology, University of Vienna, 1030 Vienna, Austria

^3^ College of Science and Engineering, Flinders University, Bedford Park, SA, 5042 Australia

^4^ Charles Darwin Research Station, Charles Darwin Foundation, Puerto Ayora, Santa Cruz, Galápagos, Ecuador

**Corresponding author:** Lauren K. Common – lauren.common@univie.ac.at

**Table S1.** Densities of natural fibres used to stock treated material dispensers on Floreana Island, taken from Mauchamp-Fessl (2024).

| **Material Type** | **Density (g/cm^3^)** |
| --- | --- |
| Sisal fibres | 1.45 |
| Cotton fibres | 1.54 |
| Kapok | 0.30 |
| Chicken feathers | 0.68 |
| Hemp fibres | 1.20 |

**Table S2.** Output from four principal component analyses, showing the eigenvalues and factor loadings for the first principal component in each analysis. PC_Neophilia is a measure of neophilia in response to a novel object, while PC_Aggressive is a measure of aggressiveness in response to a simulated territory intrusion.

| **Principal Component** | **Eigenvalue** | **Factor** | **Loading** |
| --- | --- | --- | --- |
| PC_Neophilia | 1.82 | Latency to 3 m | -0.707 |
|  |  | Minimum distance | -0.707 |
| PC_Aggressive | 3.16 | Time within 5 m | 0.461 |
|  |  | Time within 1 m | 0.388 |
|  |  | Number of flights | -0.524 |
|  |  | Number of crosses | 0.455 |
|  |  | Minimum distance | 0.393 |

**Table S3.** Output from an LMM testing the effects of sex, breeding stage, site, species, trial number, and novel object type on neophilia (response to novel object, PC_Neophilia) in Darwin’s finches on Floreana Island. The random effect of nest ID explained 0.61 ± 0.78 of the variance in the data. Species abbreviations: SGF = small ground finch, STF = small tree finch, MTF = medium tree finch, CF = cactus finch. Bold values indicate statistical significance P < 0.05.

|  | **Estimate** | **SE** | **t-value** | **χ^2^** | **df** | **P** |
| --- | --- | --- | --- | --- | --- | --- |
| Intercept | 0.21 | 1.02 | 0.21 |  |  |  |
| Sex [Male]* | 0.12 | 0.32 | 0.38 | 0.18 | 1 | 0.701 |
| Stage [Paired] ** | 0.25 | 0.37 | 0.67 | 0.45 | 1 | 0.501 |
| Site [Cerro Pajas]*** | 0.28 | 0.31 | 0.89 | 1.70 | 3 | 0.636 |
| Site [Lowlands]*** | 0.75 | 0.69 | 1.09 |  |  |  |
| Site [Town]*** | 0.91 | 1.43 | 0.64 |  |  |  |
| Species [MTF]^ | -0.37 | 0.92 | -0.40 | 1.73 | 3 | 0.631 |
| Species [SGF]^ | -0.76 | 0.85 | -0.89 |  |  |  |
| Species [STF]^ | -0.63 | 0.89 | -0.71 |  |  |  |
| Novel object [2]^^ | 0.13 | 0.38 | 0.35 | 6.21 | 3 | 0.102 |
| Novel object [3]^^ | -0.01 | 0.39 | -0.04 |  |  |  |
| Novel object [4]^^ | -1.23 | 0.58 | -2.11 |  |  |  |

Reference categories set to: * Female, ** Defending, *** Asilo de la Paz, ^ CF, ^^ novel object 1

**Table S4.** Output from an LM testing the effects of breeding stage, site, and species on aggressiveness (response to simulated territory intrusion, PC_Aggressive) in Darwin’s finches on Floreana Island. Species abbreviations: SGF = small ground finch, STF = small tree finch, MTF = medium tree finch, CF = cactus finch. Bold values indicate statistical significance P < 0.05.

|  | **Estimate** | **SE** | **t-value** | **SumSq** | **F-Value** | **P** |
| --- | --- | --- | --- | --- | --- | --- |
| Intercept | 0.54 | 1.13 | 0.48 |  |  |  |
| Stage [Paired] * | -0.14 | 0.53 | -0.26 | 0.18 | 0.07 | 0.796 |
| Site [Cerro Pajas]** | -0.19 | 0.50 | -0.38 | 0.58 | 0.11 | 0.897 |
| Site [Lowlands]** | 0.08 | 0.83 | 0.10 |  |  |  |
| Species [MTF]^ | -1.07 | 1.11 | -0.96 | 31.61 | 4.00 | **0.012** |
| Species [SGF]^ | 0.73 | 0.95 | 0.76 |  |  |  |
| Species [STF]^ | -1.26 | 1.10 | -1.15 |  |  |  |

Reference categories set to: * Defending, ** Asilo de la Paz, ^ CF.


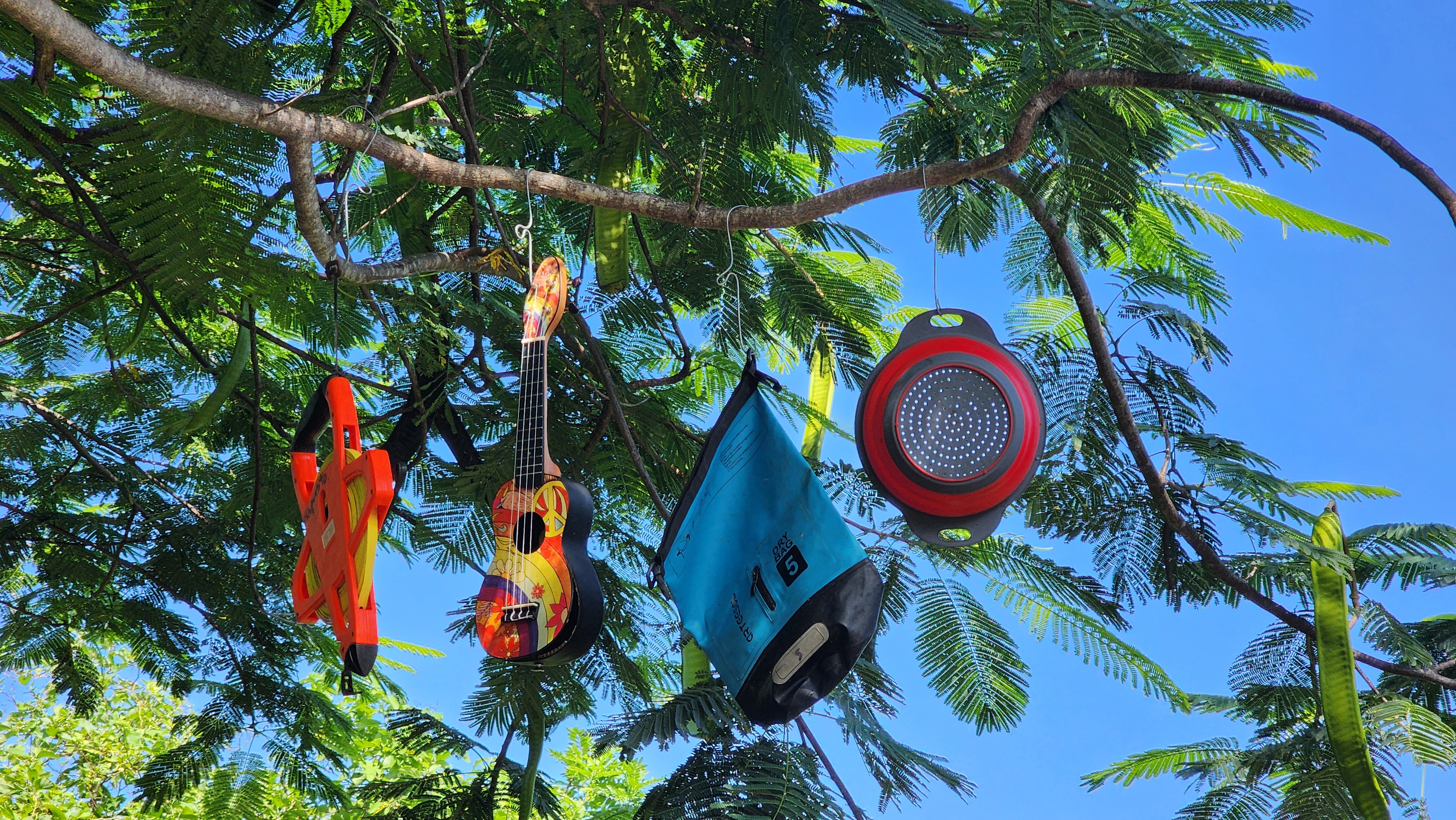


**Figure S1.** The novel objects used for our novel object trials: (from left to right) tape measure (novel object 3), ukulele (novel object 4), blue dry bag (novel object 1), and expandable strainer (novel object 2).


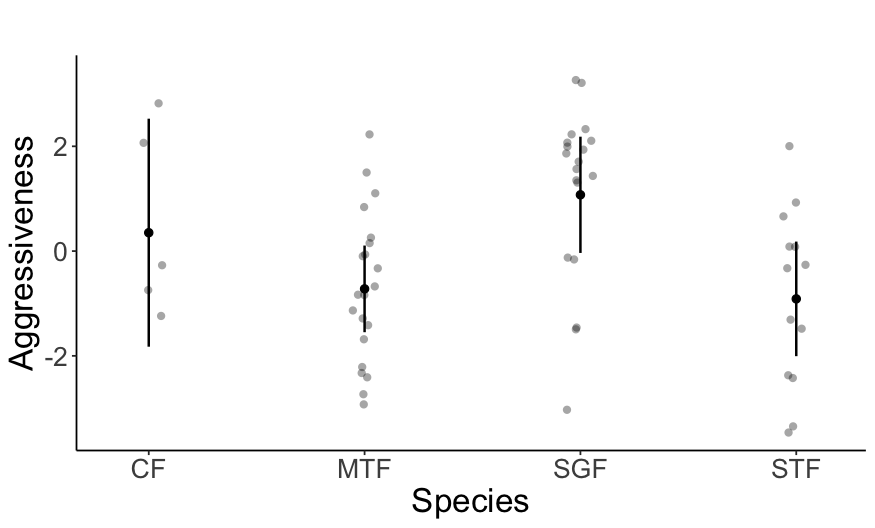


**Figure S2.** Species differences in aggressiveness (response to simulated territory intrusion, PC_Aggressive) in Darwin’s finches on Floreana Island. Higher scores for PC_Aggressive indicate higher aggressiveness, i.e., more time spent within 5 m and 1 m of the speaker, shorter minimum distance, and more flights and crosses. Raw data are presented as circles. Full model output is presented in Table S3. Species abbreviations: SGF = small ground finch, STF = small tree finch, MTF = medium tree finch, CF = cactus finch.
